# Multivariate Design of 3D Printed Immediate-Release Tablets with Liquid Crystal-Forming Drug—Itraconazole

**DOI:** 10.3390/ma13214961

**Published:** 2020-11-04

**Authors:** Witold Jamróz, Jolanta Pyteraf, Mateusz Kurek, Justyna Knapik-Kowalczuk, Joanna Szafraniec-Szczęsny, Karolina Jurkiewicz, Bartosz Leszczyński, Andrzej Wróbel, Marian Paluch, Renata Jachowicz

**Affiliations:** 1 Department of Pharmaceutical Technology and Biopharmaceutics, Jagiellonian University Medical College, Medyczna 9, 30-688 Krakow, Poland; jolanta.pyteraf@uj.edu.pl (J.P.); joanna.szafraniec@uj.edu.pl (J.S.-S.); renata.jachowicz@uj.edu.pl (R.J.); 2 Division of Biophysics and Molecular Physics, Institute of Physics, University of Silesia, Uniwersytecka 4, 40-007 Katowice, Poland; justyna.knapik-kowalczuk@us.edu.pl (J.K.-K.); karolina.jurkiewicz@us.edu.pl (K.J.); marian.paluch@us.edu.pl (M.P.); 3 Silesian Center for Education and Interdisciplinary Research, University of Silesia, 75 Pulku Piechoty 1a, 41-500 Chorzow, Poland; 4 Marian Smoluchowski Institute of Physics, Jagiellonian University, Łojasiewicza 11, 30-348 Krakow, Poland; bartosz.leszczynski@uj.edu.pl (B.L.); andrzej.wrobel@uj.edu.pl (A.W.)

**Keywords:** 3D printing, fused deposition modeling, hot-melt extrusion, solid dosage forms, itraconazole

## Abstract

The simplicity of object shape and composition modification make additive manufacturing a great option for customized dosage form production. To achieve this goal, the correlation between structural and functional attributes of the printed objects needs to be analyzed. So far, it has not been deeply investigated in 3D printing-related papers. The aim of our study was to modify the functionalities of printed tablets containing liquid crystal-forming drug itraconazole by introducing polyvinylpyrrolidone-based polymers into the filament-forming matrices composed predominantly of poly(vinyl alcohol). The effect of the molecular reorganization of the drug and improved tablets’ disintegration was analyzed in terms of itraconazole dissolution. Micro-computed tomography was applied to analyze how the design of a printed object (in this case, a degree of an infill) affects its reproducibility during printing. It was also used to analyze the structure of the printed dosage forms. The results indicated that the improved disintegration obtained due to the use of Kollidon^®^CL-M was more beneficial for the dissolution of itraconazole than the molecular rearrangement and liquid crystal phase transitions. The lower infill density favored faster dissolution of the drug from printed tablets. However, it negatively affected the reproducibility of the 3D printed object.

## 1. Introduction

Additive manufacturing has huge potential to revolutionize the methods of drug delivery system formation. It was proven for mass-scale drug production by Aprecia Pharmaceuticals, which registered the first 3D printed drug, Spritam^®^, in 2015. However, the use of additive manufacturing also enables the preparation of small batches of customized, on-demand-prepared formulations—for example, in the treatment of patients with rare diseases or for clinical trials. The great applicability of 3D printing (3DP) in the pharmaceutical field results from the simplicity of object shape modification, which allows the production of dosage forms of complex shape and internal structure, containing one or more active pharmaceutical ingredients (APIs) [1,2]. Moreover, the differences in shape and infill density of tablets, which cannot be achieved in compressed tablets, lead to alternation in the surface-to-volume ratio and allow us to produce printlets with desired drug dosages and dissolution profiles [3,4,5]. Although the issue of the correlation between the internal structure of printed tablets and their properties, particularly the dissolution characteristics, has been explored by several research teams, there is still deficiency in studies on the actual microstructure and quality of printed objects and the mechanisms driving the release of the drug from printed dosage forms [6,7,8,9].

In the case of nearly all 3DP methods, the object is built layer by layer based on the computer aided design (CAD) model. However, various printing technologies vary between each other regarding used materials and process conditions such as temperature. The 3D printing methods can operate with a powder, which is bound with a liquid binder or sintered with a laser, a photosensitive resin, a thermoplastic material, or a semi-solid formulation extruded through the printer nozzle. Several techniques, such as stereolithography [10,11,12,13,14], selective laser sintering [8,15] digital light processing [16,17], binder jetting, [18,19], and extrusion-based methods including direct powder extrusion [20,21], semi-solid extrusion [22,23,24,25,26], and fused deposition modeling (FDM) [27,28], have been investigated for application in the pharmaceutical industry. The 3DP methods which can be introduced in the high-scale manufacturing process should be characterized by the high-speed production of uniform objects [29,30]. In the case of most of the abovementioned printing methods, process conditions may cause amorphization of the active ingredient, which increases its solubility [31,32].

Various dosage forms, such as orodispersible films [33], mucoadhesive films [34], immediate and modified-release tablets [35,36], capsules [37,38], implants [39], or even formulations imitating sweets [40], have been recently developed using fused deposition modeling. In the printed dosage forms, drug release modification is obtained mostly by selecting either the filament-forming polymers characterized by suitable pH-dependent solubility [41] or the printlet shape and geometry, i.e., the presence of channels [42], empty cavities (floating tablets) [43,44], variations in the infill degree or shape as well as the use of shape-memory polymers to prepare retentive drug delivery systems (4D printing) [45,46]. Despite the fabrication of dosage forms by means of 3DP, this technique can be used for capsular shell fabrication [47] to control the API’s dissolution process as well as mold preparation to create custom-made, patient-oriented drugs [48]. The 3D printed molds can be also used in a range of science and technology sectors including electrochemical electrical applications—for example, flexible sensor prototypes [49,50].

The application of FDM printing technology in the manufacturing of dosage forms requires the use of previously prepared drug-loaded filament. Filaments are produced mostly in the hot-melt extrusion process (HME), which is also the method applied to increase drug solubility. During this process, a mixture of drug and thermoplastic polymer is heated and blended, and the molten mass is pushed through a nozzle to form a filament [51]. Instead of drugs, other substances can be used in the HME process, e.g., insoluble hydroxyapatite for filament fabrication, which can be used in bone tissue engineering [52]. One of the most important advantages is that this process does not require the use of organic solvents, such as the preparation of amorphous solid dispersion (ASD) by spray drying. However, HME operates at high temperatures, which are required to melt the formulation components [53]. In some cases, it is necessary to add plasticizers to the formulation to lower the process temperature in order to protect the thermolabile active ingredient and improve filament printability [36,54,55]. The combination of HME and FDM can induce phase transitions, including amorphization, which results in increased drug solubility. Further drug dissolution modification can be also achieved by changing the shape and surface of the printed dosage form [26].

Itraconazole (ITR) is an oral antifungal agent used in the treatment of systemic and superficial fungal infections, commercially available in the form of 65 mg and 100 mg capsules, 200 mg tablets, and 10 mg/mL solutions. It is a highly lipophilic, weakly alkaline drug with very low water solubility of 1 ng/mL at pH 7 and 4 μg/mL at pH 1. ITR is classified as a Biopharmaceutics Classification System (BCS) class II substance [56], which means it has solubility-limited bioavailability. The drug exhibits three polymorphs varying in stability and solubility [57]. Moreover, ITR can form liquid crystals, which are particularly interesting from the perspective of pharmaceutical sciences. Liquid crystals can adopt various molecular arrangements (nematic and smectic in the case of ITR), which affect the free energy of the system and thus the dissolution performance. Due to the relatively high glass transition temperature (T_g_ = 59 °C), ITR can be also transformed into a stable amorphous state, usually in the form of amorphous solid dispersions with polymers or co-amorphous systems with small molecules [58].

Soluplus^®^ [59,60,61], Eudragit^®^ L [62], polyvinylpyrrolidone (PVP) [63], Kollidon^®^ VA64 [64,65], polyvinyl alcohol (PVA) [65,66], as well as semi-synthetic cellulose derivatives such as hydroxypropyl cellulose [67] and hydroxypropyl methylcellulose acetate succinate [53,54,68,69,70], are examples of pharmaceutical polymers tested for preparing itraconazole amorphous solid dispersions (ASD) and also suitable as filament-forming polymers for FDM. Although many papers described the formation of amorphous solid dispersions with ITR, including the use of the hot-melt extrusion process [61], only two considered the formation of dosage forms using 3D printing. Kimura et al. reported that it is possible to use fused deposition modeling to prepare zero-order sustained-release floating tablets containing itraconazole [43]. They were able to control floating time by printing tablets with empty cavities inside and to modify the drug dissolution rate by changing the tablet surface and wall thickness. Goyanes et al. prepared tablets containing amorphous solid dispersions of itraconazole in different grades of hydroxypropylcellulose using direct powder extrusion 3D printing—a novel, single-step 3D printing process. In contrast to FDM, this 3D printer tool head is equipped with single screw extruder, which allows it to print directly using mixed powders or pellets, without preparing filaments [20].

In this paper, we describe for the first time the liquid crystal phase transitions of itraconazole in 3D printed tablets. The drug was combined with polymers, formed into filaments via hot-melt extrusion and then printed using fused deposition modeling technology. The filaments were based on poly(vinyl alcohol), a water-soluble semi-crystalline polymer known for its superior printability. The two PVP-based polymers were also added to the filament-forming mixture to introduce the additional functionalities into the printed matrices. Kollidon^®^ VA64 was supposed to modify the physicochemical properties—the molecular arrangement in particular (analyzed using thermal analysis and X-ray diffractometry)—and Kollidon^®^ CL-M was added to modify drug dissolution due to the improved tablet disintegration. We performed deep micro-computed tomography (µ-CT) analysis as the first attempt to analyze how the design of a printed object (degree of an infill) affects its reproducibility during printing. It was also used to analyze the structure of the printed dosage forms to support the dissolution data. To clearly understand the advantages of extrusion and printing processes, drug dissolution from printed formulations was compared with tablets having similar composition, obtained by the compression of either raw powders or milled filament.

## 2. Materials and Methods

### 2.1. Materials

Itraconazole (ITR, 1-(butan-2-yl)-4-{4-[4-(4-{[(2R,4S)-2-(2,4-dichlorophenyl)-2-[(1H-1,2,4-triazol-1-yl)methyl]-1,3-dioxolan-4-yl]methoxy}phenyl)piperazin-1-yl]phenyl}-4,5-dihydro-1H-1,2,4-triazol-5-one, 99.8%, Henan Tianfu Chemical Co., Ltd., Zhengzhou, China) served as a model drug. Poly(vinyl alcohol) (PVA, Parteck^®^ MXP, Merck^®-^ KGaA, Darmstadt, Germany), copovidone (K/VA, Kollidon^®^ VA64, BASF^®^, Ludwigshafen, Germany), crospovidone (K/CL, Kollidon^®^ CL-M, BASF^®^, Ludwigshafen, Germany) were utilized as the matrix-forming polymers to prepare both filaments and 3D printed tablets. Talc (Fagron^®^, Kraków, Poland) and magnesium stearate (Avantor^®^ Performance Materials, Gliwice, Poland) were added to tablets prepared by compression in tablet press. Hydrochloric acid (Merck^®^ KGaA, Darmstadt, Germany) and potassium chloride (Avantor^®^ Performance Materials, Gliwice, Poland) were used as dissolution media ingredients. Water used in all experiments was produced by Elix 15UV Essential reversed osmosis system (Merck^®^ KGaA, Darmstadt, Germany).

### 2.2. Preparation of Drug-Loaded Filaments

**Figure 1:**
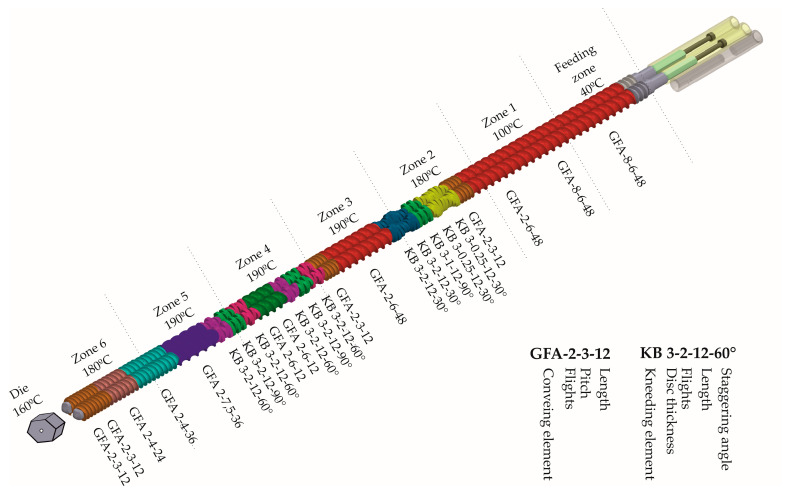
Screw configuration and temperature profile.

Filaments were extruded using a 40D, 12-mm co-rotating twin-screw extruder (RES-2P/12A Explorer, Zamak Mercator^®^, Skawina, Poland) equipped with a gravimetric feeder MCPOWDER^®^ (Movacolor^®^, Sneek, The Netherlands) and an air-cooled conveying belt (Zamak Mercator^®^, Skawina, Poland). The mixtures of itraconazole and matrix-forming polymers, of the composition presented in Table 1, and the total mass equal to 200 g were extruded through a 1.75 mm die at 160 °C. The feeding rate was set to approximately 70 g/h, which resulted in the linear filament extrusion speed of 25 m/h. The barrel temperature varied from 40 to 190 °C. The optimized temperature profile and screw configuration are presented in Figure 1.

### 2.3. Evaluation of Filament Properties

The diameter uniformity of the obtained filament was evaluated using a Mitutoyo^®^ micrometer screw (Kawasaki, Japan). Six randomly selected points were measured. Mechanical properties were assessed in stretching test performed with an EZ-SX tensile tester (Shimadzu^®^, Kioto, Japan). The measurements were performed six times for each type of filament. Randomly selected pieces of filament, 100 mm in length, were placed in the tensile tester’s jaws and stretched up to breakage. Hardness and elasticity of the filaments were determined based on the measurements of tensile strength and Young’s modulus.

### 2.4. Determination of Itraconazole Content in the Obtained Filament

Six randomly selected and accurately weighed pieces of filament were placed in conical flasks filled with 25 mL of a mixture of methanol and 0.1 M HCl of pH 1.2 (1:1 v/v) and shaken for 24 h using a Memmert^®^ water bath (WNB 22, Schwabach, Germany). The drug concentration was assayed at λ = 255 nm using a Shimadzu^®^ UV-1800 spectrophotometer (Kioto, Japan). The specificity of the analytical method was verified. There was no sign of interference between the drug and excipients at the analytical wavelength.

### 2.5. Preparation of 3D Printed Tablets

The Blender^®^ 2.79b software was used to design the models of the oblong tablets (Blender Foundation, Amsterdam, The Netherlands). The basic model was 20 mm long and 10 mm wide. The model height varied from 2.4 to 3.65 mm, which was related to the number of 3D printed layers. Voxelizer^®^ slicing software (version 1.4.18, ZMorph^®^, Wroclaw, Poland) was applied to define the height and the width of the single layer path. The 3D model was imported in stl format and divided into layers before printing. The thickness of the first layer was equal to 0.2 mm to improve the adhesion of the print to the printer bed, whereas the height of the subsequent layers was 0.15 mm. The path width was equal to the diameter of the printing nozzle, i.e., 0.4 mm. One outline and rectilinear infill (density of 20%, 35%, and 60%) were designed for the printing process. Each tablet was composed of 50 mg of ITR and 200 mg of polymer carriers (Table 1). The tablets were printed by an FDM ZMorph^®^ 2.0 S personal fabricator (Wroclaw, Poland) equipped with a 1.75 mm commercially available printhead. Printing temperature was 205 °C. The tablets were printed with a 10–15 mm/s printing speed. The temperature of building platform was 40 °C.

### 2.6. Preparation of Tablets by Filament Compression (HME Tablets)

For comparison purposes, filament milled in a Tube Mill 100 control (IKA^®^, Staufen, Germany) and raw compounds were compressed in a Korsch^®^ EK0 single-punch tablet press (Berlin, Germany). The composition of the tablets was similar to 3D printed tablets; each tablet was composed of 50 mg of ITR and 200 mg of polymer mixture. Additionally, the blends contained 12.5 mg of a talc and magnesium stearate mixture (9:1 w/w), which played the role of glidant and lubricant, respectively.

### 2.7. Preparation of Directly Compressed Tablets (DC Tablets)

Powder blends composed of 3DP tablet ingredients with the addition of the talc and magnesium stearate mixture (9:1 w/w) were compressed using Korsch^®^ EK0 single-punch tablet press (Berlin, Germany) for comparison purposes, to investigate the impact of technological processes on the ITR dissolution profile.

### 2.8. Micro-Computed Tomography

Micro-computed tomography (µ-CT) analysis was performed using a SkyScan^®^ 1172 microtomograph (Bruker^®^, Billerica, MA, USA). It was applied to examine the structure of the 3DP tablets with 20%, 35%, and 60% of infill and to verify the repeatability of printing process (the data collected for three tablets with 35% of infill were compared). The image pixel size was 6.9 µm for measurements of all samples. A cone beam reconstruction software program (Nrecon SkyScan^®^, Bruker^®^, Billerica, MA, USA) based on the Feldkamp algorithm was used for the reconstruction of the projections. A CT-Analyser^®^ (SkyScan^®^, Bruker^®^, Billerica, MA, USA) was used for binarization purposes. The procedure was based on density distribution histograms collected for the whole sample volume. A CT-Analyser^®^ was also used for the characterization of the morphological features of the tablets, their volume, and surface. CTVox^®^ software (Bruker^®^, Billerica, MA, USA) was applied to present the 3D results.

### 2.9. Differential Scanning Calorimetry (DSC)

Thermodynamic properties of neat ITR, PVA, K/VA, K/CL, and their mixtures in the form of filaments and 3DP tablets were examined using a DSC 1 STAR^e^ System (Mettler-Toledo^®^, Greifensee, Switzerland) equipped with an HSS8 ceramic sensor with 120 thermocouples and liquid nitrogen cooling station. Zinc and indium standards were used for the temperature and enthalpy calibration. The samples were measured in an aluminum, pinned crucible (40 mL). The samples were heated with a rate of 10 K/min. The experiments were performed in nitrogen atmosphere with a gas flow of 60 mL/min.

### 2.10. X-Ray Powder Diffraction (XRD)

A Rigaku Denki^®^ D/MAX Rapid II-R (Tokyo, Japan) equipped with a rotating Ag anode and an image plate detector in the Debye–Scherrer geometry was used for the X-ray diffraction measurements. Graphite (002) crystal was used to monochromatize the incident radiation (λ_Kα_ = 0.5608 Å). The width of the X-ray beam at the sample was 0.3 mm. The samples were pulverized before the experiment and measured at room temperature, in glass capillaries with a diameter of 1.5 mm and wall thickness of 0.01 mm. The background intensity from empty capillary was subtracted. The obtained two-dimensional diffraction patterns were converted into one-dimensional functions of intensity versus the scattering vector.

### 2.11. Dissolution Studies

The dissolution of ITR from tablets was determined in 1000 mL of 0.1 M HCl with the addition of KCl, in the pharmacopeial paddle apparatus (Vision^®^ G2 Elite 8, Hanson Research^®^, Chatsworth, CA, USA) equipped with a VisionG2 AutoPlus autosampler. Stainless steel, spring-like sinkers were used to prevent tablet floating. The samples were filtered and analyzed on-line at 255 nm at predetermined periods using a UV-1800 spectrophotometer (Shimadzu^®^, Kioto, Japan) equipped with flow-through cuvettes. Three repetitions for each sample were carried out. The results represent the averaged results and the standard deviations (mean ± SD).

### 2.12. Solubility Study

An excess of physical mixture (PM), extrudate (HME), and printed systems (3DP) were dispersed in 20 mL of 0.1 MHCl and shaken at ambient temperature using a KS 130 basic orbital shaker (IKA^®^, Staufen im Breisgau, Germany). After 48h, the samples were filtered through a 0.45 µm Chromafil^®^ Xtra CA-45/25 membrane filter and analyzed spectrophotometrically at λ = 255 nm (UV-1800 Shimadzu^®^, Kioto, Japan). The reported data represent the averages from three series of measurements with standard deviations (SD).

## 3. Results

### 3.1. Evaluation of the Filaments

All prepared filaments were made using a PVA as a filament-forming polymer, a semi-crystalline polymer of molecular weight equal to 32 kDa with 87–89% hydrolysis grade, having a glass transition temperature, melting point, and degradation temperature of 40–45 °C, 170 °C, and ≥250 °C, respectively [66]. The obtained itraconazole-loaded filaments were opaque and creamy in color. The diameter of the filaments was kept at a constant level; however, in the case of the PVA_K/CL filament, the diameter variations were higher than 0.05 mm, which is considered as a maximum acceptable deviation from the declared diameter [71]. The itraconazole content and its uniformity were satisfactory. All the API-loaded filaments were tested for their tensile strength and elasticity, which were found to be critical quality attributes in term of printability. The results are presented in Table 2. It was found that the addition of copovidone and crosslinked PVP resulted in a decrease in the tensile strength and Young’s modulus of the filaments. All the prepared filaments were able to be printed with a ZMorph^®^ 2.0 S 3D printer immediately after extrusion and after storage in zipper storage bags.

**Figure 2:**
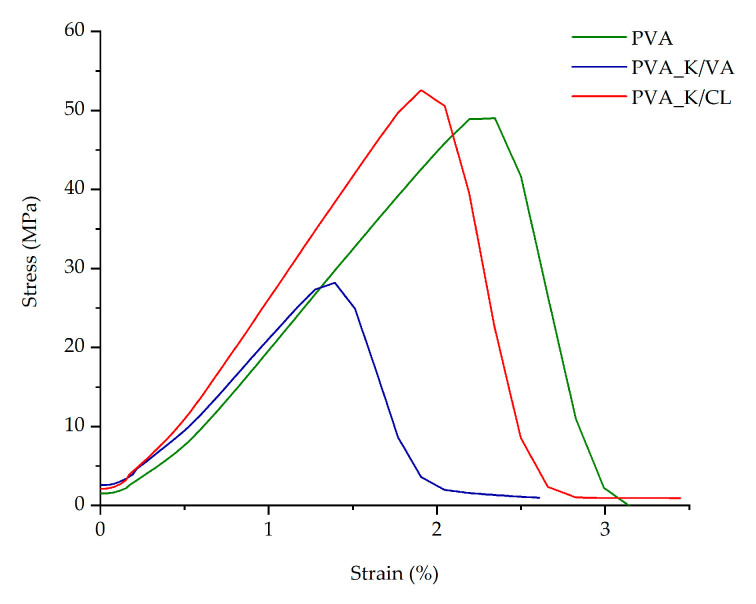
Comparison of the mechanical strength and resilience of the filaments.

In Figure 2, the differences in the mechanical characteristics are presented. The Young’s modulus corresponds to the slope of the curve in the elastic behavior region.

### 3.2. Thermal and Structural Properties of the Filaments and 3DP Tablets

**Figure 3:**
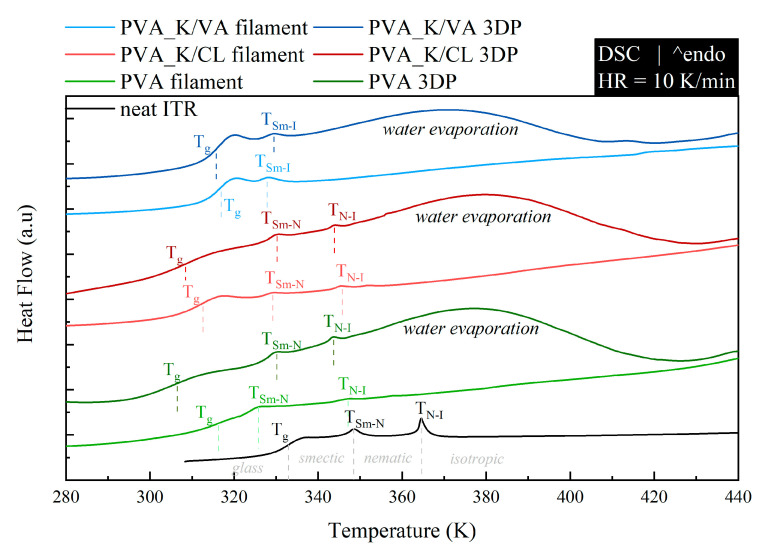
DSC thermograms of neat ITR and its mixtures with PVA, PVA_K/VA, and PVA_K/CL prepared in two forms: filament and 3DP tablet.

To investigate how the employed polymers modify the thermal properties of neat ITR, the systems, prepared in the form of both filaments and 3DP tablets, were measured (after pulverization) by means of DSC. The samples were examined in the temperature range from 273 to 453 K at a heating rate of 10 K/min. In Figure 3, the obtained DSC traces are compared to the thermogram of the neat, quench-cooled ITR. Because the used PVA polymer has a lower glass transition temperature than ITR (Table 3) (T_g_ of neat PVA and ITR are equal to 313 and 332 K, respectively), the plasticization effect was observed. Interestingly, the DSC thermograms of the same compositions with different forms, filament or 3DP, differ from each other. As can be seen in Figure 3, the thermograms of the 3DP tablets are characterized by: (i) a shift in glass transition temperature towards lower values when compared with filament and (ii) the appearance of an additional, very broad endothermal event in the vicinity of 320 to 420 K. The observed differences suggest that the 3DP tablets also contain water in addition to API and polymers. Water exerts a plasticization effect on the samples and evaporates at temperatures from in the range of 320 to 420 K.

When the neat ITR is heated above its glass transition temperature, on the DSC thermogram, one can distinguish two endothermal processes associated with the liquid crystal (LC) phase transitions. The thermal event located at 348 K reflects transition from smectic (Sm) to nematic (N) LC alignment, while at 364 K, ITR loses the nematic order and becomes an isotropic (I) liquid. The performed experiments reveal that the employed polymers shift to lower temperatures for both Sm-N and N-I phase transition. The determined, based on calorimetric studies, values of T_g_, T_Sm-N_, and T_N-I_ for all investigated systems are compared in Table 3. It is worth noting that in one of the examined systems (PVA_K/VA), regardless of the applied technological process, the lack of the nematic phase was observed (i.e., the N-I endothermal event was not registered by means of DSC).

**Figure 4:**
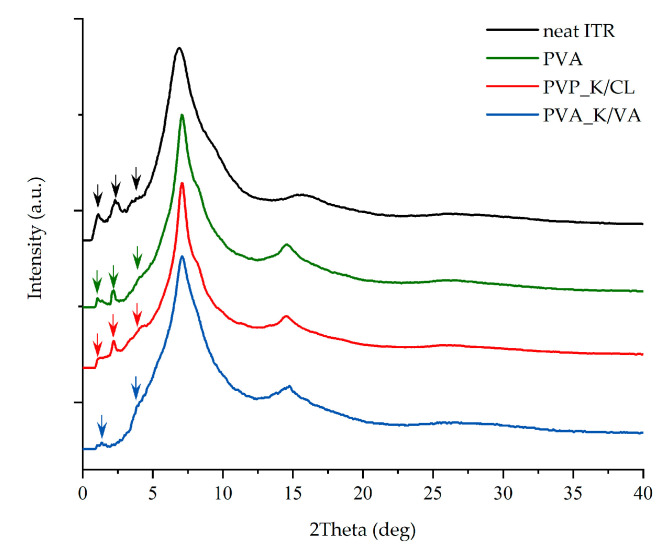
XRD diffraction patterns of neat ITR and its mixtures with PVA, PVA_K/VA, and PVA_K/CL in an initial form of 3DP tablet.

In order to investigate whether the employed polymers indeed modify the ITR’s LC alignment, both the neat ITR as well as the pulverized 3DP tablets were measured by wide-angle X-ray diffraction (XRD) technique. The comparison of the scattering patterns collected at room temperature for neat ITR and pulverized tablets containing either PVA, PVA_K/VA, or PVA_K/CL is presented in Figure 4. The presented XRD patterns demonstrate that the polymers affect the LC order in ITR. As can be seen, samples containing PVA or PVA_K/CL reveal less intense peaks at around 0.22, 0.45, and 0.68 Å^−1^, which are indicators of smectic layering [72]. In the case of the system containing K/VA, the reduction in the intensity of the peaks at 0.22 and 0.68 Å^−1^ is combined with the disappearance of the peak at 0.45 Å^−1^. These results indicate that the layered structure in ITR is medicated by the employed additives.

### 3.3. Micro-Computed Tomography Studies of Tablets

The dimensions and masses of 3DP tablets corresponded to predefined values. The average tablet mass ranged from 239.73 to 253.05 mg. Tablet length varied from 19.85 to 20.15 mm, whereas height ranged from 1.78 to 3.65 mm. The real layer height was from 0.142 to 0.158 mm and was calculated by dividing the tablet height by the number of layers, given the fact that the first layer was 0.2 mm (Table 4). Digital photos of 3D printed tablets can be found in the Supplementary Materials associated with this article (Figures S1–S3, Supplementary Materials).

**Figure 5:**
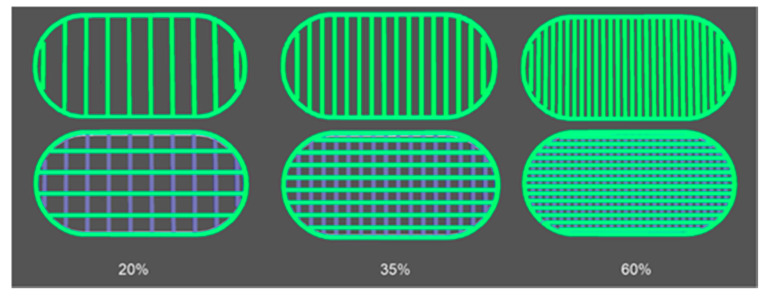
Images of PVA_K/CL tablet layers obtained from Voxelizer software.

Based on the 3D tablet images obtained from Voxelizer slicing software (Figure 5) and predefined settings of the path size, the theoretical volume of 3DP PVA_K/CL tablets was calculated. The values varied from 184.4 mm^3^ for T_20 tablets to 195.6 mm^3^ for T_60 and 195.9 mm^3^ for T_35 tablets.

**Figure 6:**
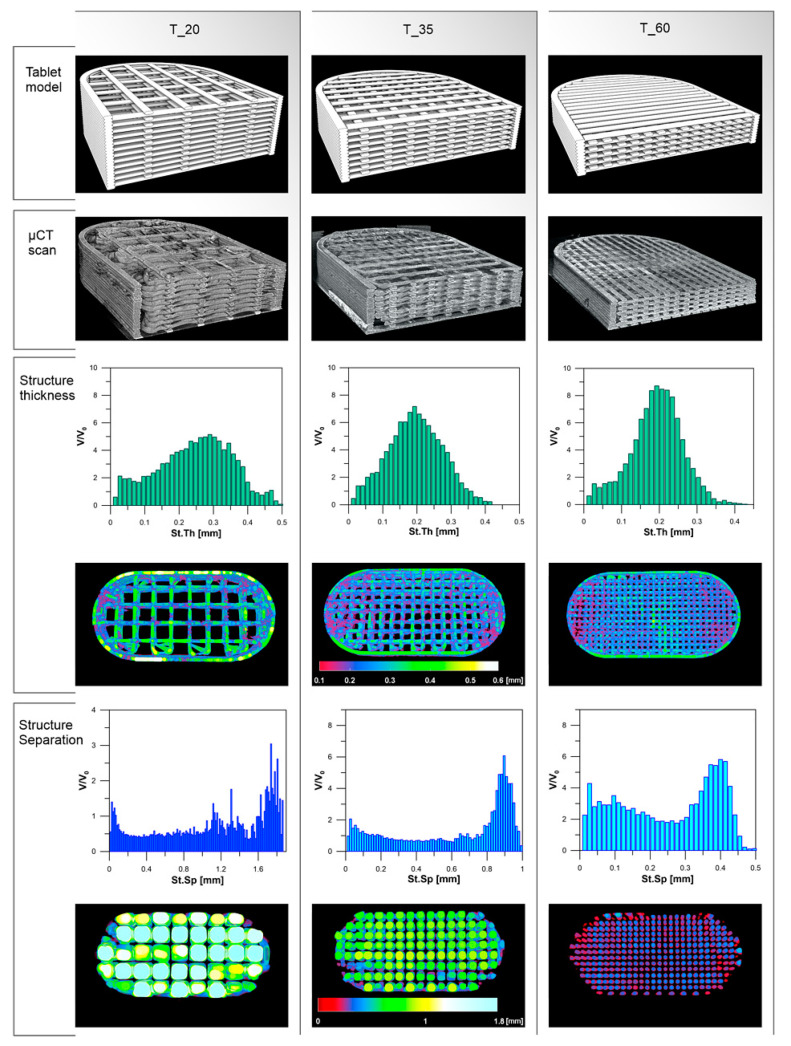
3D tablet models, µ-CT scan images of 3DP tablets, and structure thickness and structure separation of 3DP tablets.

The morphology of the PVA_K/CL printed tablets was verified by the µCT scans. Tablets with 20% of infill had the highest object volume (236 mm^3^) and the highest open pore volume (485 mm^3^). Medium pore size (structure separation) was 1.11 mm, whereas the average structure thickness was 0.25 mm. Tablets with 60% of infill were characterized by the lowest values of object volume (202 mm^3^) and pore volume (134 mm^3^) as well as structure separation (0.19 mm) and pore size (0.25 mm). Aforementioned parameters for tablets with 35% of infill can be placed between T_20 and T_60 values (Table 5, Figure 6).

**Figure 7:**
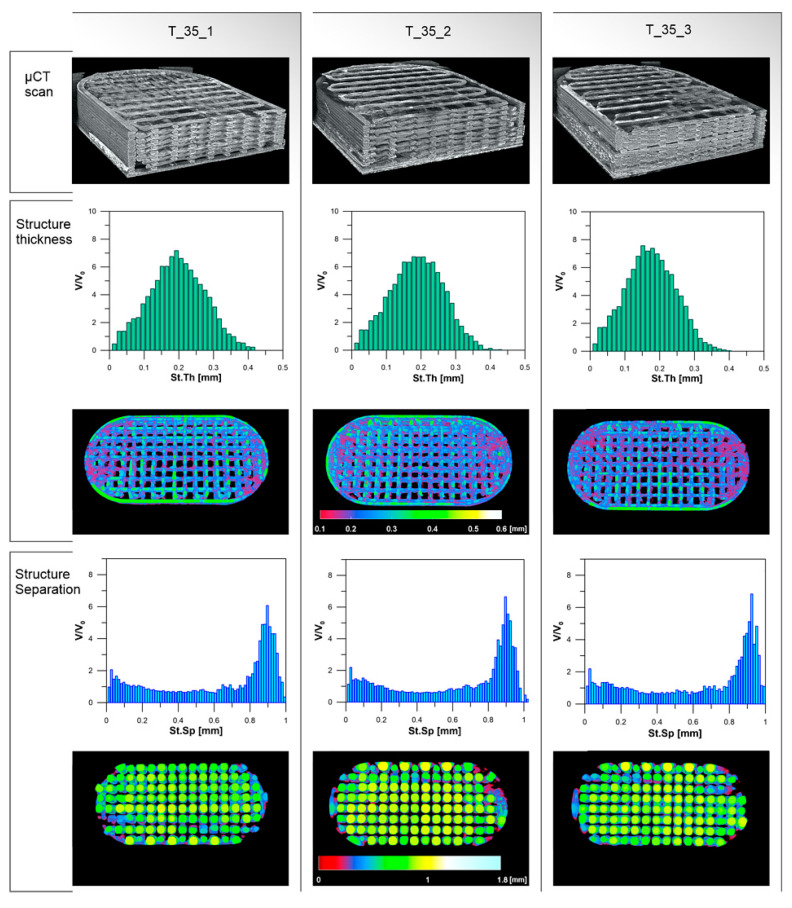
Comparison of 3DP tablets with 35% of infill.

Parameters of tablets with 35% of infill are similar and no important differences between the three analyzed tablets can be distinguished (Table 6, Figure 7).

### 3.4. Dissolution Studies

**Figure 8:**
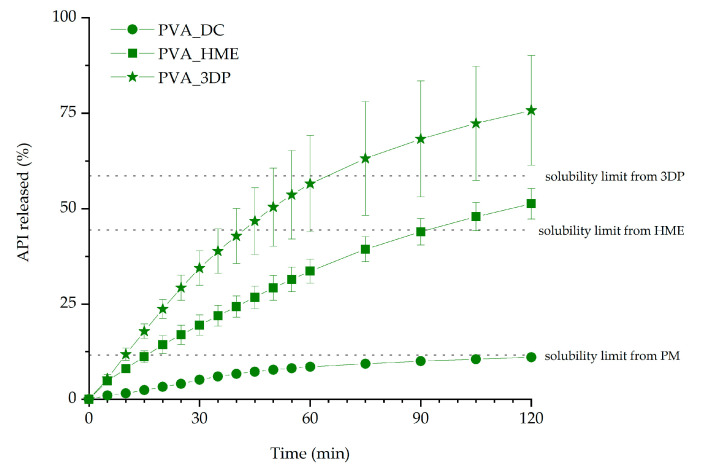
The influence of the technological process on the release profiles of itraconazole from PVA-based tablets (infill density equal to 35%).

Itraconazole dissolution from 3D printed tablets with 35% infill was compared with the dissolution profiles obtained for the tablets made from milled extrudate (HME tablets) and directly compressed tablets (DC tablets) to evaluate the impact of the excipients and hot-melt extrusion on the dissolution of the API. Determined itraconazole solubility limits were equal to 5.8, 22.2, and 29.3 µg/mL for physical mixture, extrudate, and 3D printed matrix, respectively. The solubility limits were calculated as the percentage of ITR dose in tablets (11.6%, 44.4%, and 58.6% for physical mixture, extrudate, and 3D printed tablet, respectively) and are marked in Figure 8 to make the interpretation of the dissolution easier. It was found that the performed technological processes, namely hot-melt extrusion and 3D printing, affected the dissolution profile of itraconazole. The highest amount of the drug was dissolved from 3D printed tablets. The amount of ITR released from milled extrudate was significantly lower, while the smallest amount was released from directly compressed tablets (Figure 8). After 2 h of the dissolution test, 75.8%, 51.3%, and 11.0% of the itraconazole was released from the PVA-based 3D printed, hot-melt extruded, and directly compressed tablets, respectively. This relationship was confirmed for all the prepared formulations. It must be highlighted that in the case of all 3D printed formulations, i.e., PVA, PVA_K/VA, and PVA_K/CL, the amount of dissolved itraconazole was far above the solubility limit and the supersaturation lasted as long as the dissolution test was performed.

**Figure 9:**
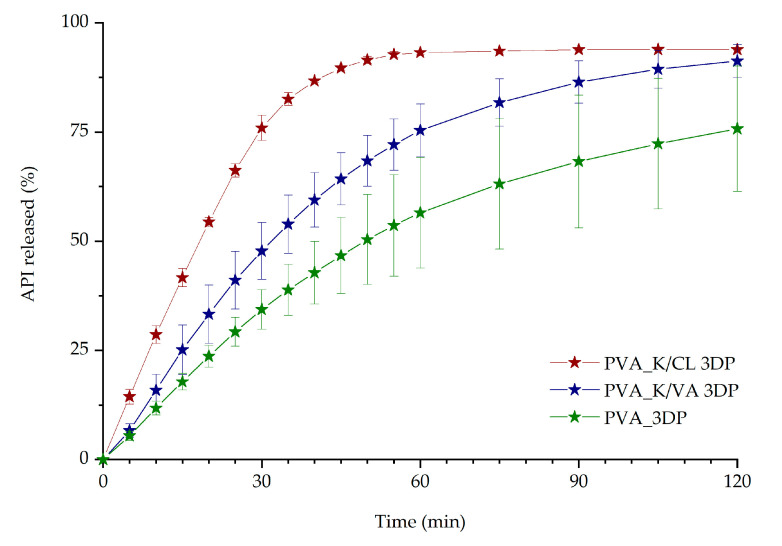
The influence of the excipients on dissolution profiles of itraconazole from 3DP tablets (infill density equal to 35%).

The impact of copovidone and crospovidone addition to the PVA formulation on the release profile was also evaluated (Figure 9). The best dissolution profile was noticed for PVA_K/CL 3D printed tablets. After 45 min, 91.5% of the API was dissolved from PVA_K/CL 3D printed tablets, while only 64.3% and 46.7% of the drug was released from 3D printed tablets with Kollidon^®^ VA64 and PVA-based tablets, respectively.

**Figure 10:**
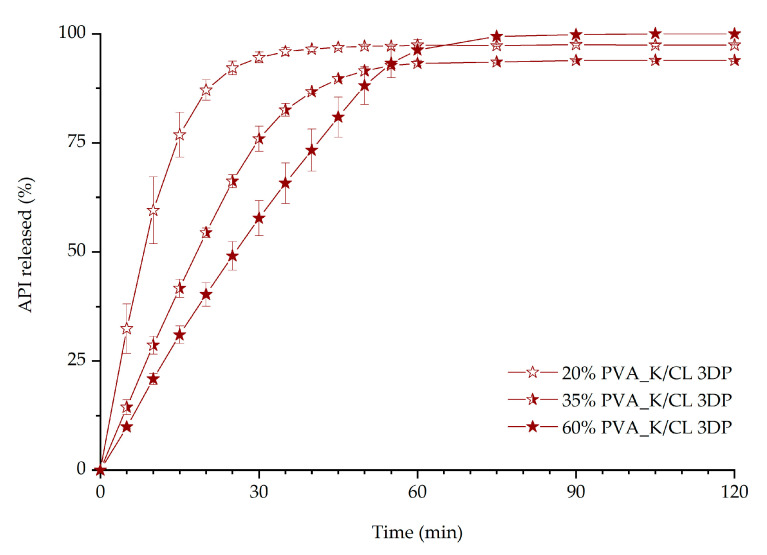
The influence of infill percentage on dissolution profiles of itraconazole from 3DP tablets.

The impact of the infill density on the dissolution characteristics was evaluated for the PVA_K/CL formulation (Figure 10) as it was selected as the most promising formulation from all the prepared 3D printed tablets. Three rectilinear infills with different densities, namely 20%, 35%, and 60%, were evaluated. The results confirmed that the lower infill density favored faster dissolution of the API. After 45 min of the dissolution test, 96.9%, 89.7%, and 80.9% of the itraconazole was released from 3D printed tablets with 20%, 35%, and 60% infill, respectively.

## 4. Discussion

The filament extrusion went smoothly, and it can be carried out as a continuous manufacturing process. As a result of the optimization of the barrel temperature profile, generated torque, which may be considered as one of the major limitations during the extrusion, was as low as 2.82 ± 0.09 Nm during the filament extrusion process. All the prepared filaments were of satisfying quality and were printable using a ZMorph^®^ 2.0 S 3D printer. PVA-based filaments were characterized by the most uniform diameter which may result from the simplest composition of the filament. Copovidone (K/VA) was added to the filament formulation to improve the solubility of the drug in the polymer matrix as it was shown by Włodarski et al. [65], while crosslinked PVP (K/CL) was added to improve disintegration and API dissolution from the extrudates and 3D printed tablets. The elasticity of the filaments was evaluated based on the Young’s modulus values. The values obtained for itraconazole-loaded filaments were in the range 2042.1–2641.1 MPa and they were comparable to the results obtained by Feuerbach et al. for Resomer filaments [73]. The filament elasticity was not significantly affected by the addition of either copovidone or crospovidone to the formulation, while the values of the Young’s modulus varied in the narrow range. However, it was found that the filament with the addition of copovidone was characterized by slightly higher elasticity than the one composed of only PVA or PVA-K/VA filaments. The obtained Young’s modulus values for all prepared filaments suggest that they are suitable for fused deposition modeling 3D printing. The tensile strength was in the range from 28.2 to 52.6 MPa; the lowest value was obtained for the filament with the addition of Kollidon^®^VA64. Its introduction to the polymer matrix caused a more than 1.7-fold decrease in tensile strength in comparison with the itraconazole-loaded PVA filament. This may result from the Kollidon^®^VA64 extrudate’s brittleness, which was confirmed by Fuenmayor et al. [74]; however, it was still durable enough to be printed.

A set of 10 × 20 mm^2^ oblong tablets with different infill densities was printed with good repeatability. The tablets were uniform in shape and mass. The dimensions of the 3DP tablets were similar to predefined 3D objects. The adjustment of tablet height and, in consequence, the number of layers was related to the filament properties to obtain tablets with comparable mass (Table 4). The differences in tablet mass did not exceed 12.5 mg (±5%) from the theoretical value of 250 mg.

The theoretical tablets’ volume was compared to the real object volume of 3DP PVA_K/CL tablets with infill of 20%, 35%, and 60% (Table 5), determined during the µCT scan. In the case of 20% of infill tablets (T_20), the real tablet volume was almost 1.3 times higher than calculated. This is related to the morphology of tablets with low infill density. The substantial distance between infill cross-points, in which two adjacent layers adhere, resulted in overhangs without support. It led to path disorder and an increase in vertical layer dimension. Therefore, subsequent cohesion in some spaces between cross-points was observed (Figure 6). In the case of T_35, the difference in tablet volume was smaller (1.12 times higher) whereas the volumes of T_60 tablets were similar (1.03 times higher). This improvement was related to the higher density of tablets’ infill with increasing number of cross-points.

The phenomenon of path expansion between the cross-points can also be explained by the deviations of the structure thickness parameter in comparison with the theoretical value of 0.15 mm. This effect was observed for all degrees of infill; however, it was less pronounced in the systems with the higher infill density (Figure 6). The biggest difference was noticed in the case of T_20 tablets, for which the mean structure thickness was 100 µm higher than the theoretical layer height. For T_35 and T_60 tablets, the structure thickness was 40–50 µm higher. The differences in the structure thickness distribution are presented in Figure 6. The widest span of structure thickness was noticed for T_20 tablets and the structures with 0.25–0.35 mm thickness had the greatest volume within 3DP objects. On the contrary, the T_60 tablets exhibited the narrowest span, with structures of thickness varying between 0.15 and 0.25 mm highly represented within the object (Figure 6). Structure thickness distribution among a set of T_35 tested tablets was similar and showed good repeatability of printed dosage forms with 35% of infill (Table 6, Figure 7). Moreover, identical mean structure separation was observed within all T_35 tablets (Table 6) The porosity within T_35 tablets was similar, and histograms of structure separation distribution revealed that pores with size 0.8–1.0 mm are highly represented (Figure 7). Decreasing the tablet infill from 35% to 20% resulted in porosity changes. Pores with larger sizes, between 1.2 and 1.75 mm, are visible on the histograms and the total porosity increased from 58.5% to 67.2%. In the case of T_60 tablets, pore size did not exceed 0.5 mm and total porosity was almost 1.7 times smaller (39.9%) than T_20 (Figure 6). It should be emphasized that the volume of the open pore space within the 3DP T_20 tablet (485 mm^3^) is twice as high as the volume of the solid part of the tablet (236 mm^3^), whereas the volume of the open pore space of T_60 (134 mm^3^) is 1.5 times smaller than the solid part of the tablet (202 mm^3^). The tablet open space will promote the penetration of dissolution media through the tablet’s internal structure and will have an impact on its disintegration and dissolution behavior. The influence of the internal structure of 3D printed objects on their properties was highlighted and widely discussed by Nazir et al. in the comprehensive review of the various 3D printed lattice and cellular structures, their advantages and limitations [75].

The results of the dissolution studies indicate that the 3D printing process improved itraconazole release when compared with tablets made by compression with either milled extrudate or a simple powder blend. This should be attributed to the developed internal structure and resulting extended surface area as well as the molecular rearrangement in the structure of API within the polymer matrix. Itraconazole release was faster from tablets containing added copovidone than PVA alone because the hot-melt extrusion and following 3D printing led to the formation of more disordered systems, which was confirmed by the lower intensity of the characteristic peaks in the XRD diffractograms and the lack of the nematic phase confirmed by DSC in the PVA_K/VA 3DP tablets. The release of itraconazole from filaments and 3D printed tablets containing only PVA was lower than from the corresponding systems containing the additive of PVP-based polymers since its structure was more ordered, as indicated by the presence of smectic and nematic domains. It is worth mentioning that the improved drug dissolution results from the applied technological processes, not just the addition of the polymers. The results of the dissolution from directly compressed tablets revealed that the presence of the polymers themselves did not enhance the dissolution of itraconazole as the amount of dissolved API did not exceed 12% of the initial dose.

The results indicated that the addition of the disintegrant, i.e., crospovidone, to the 3D printed tablets is beneficial in terms of ITR dissolution. The addition of the disintegrant to the formulation led to a higher increase in API dissolution than adding a copovidone to achieve molecularly disordered material. With the presented results, we have demonstrated that the PVA_K/CL formulation is the most promising in terms of immediate-release tablet preparation, as it is characterized by the best dissolution profile. Subsequent optimization was performed to evaluate the possibility of further improvement of itraconazole release. The optimization included changes in the infill density, as it was confirmed by many research groups that infill density significantly affects the dissolution rate of the API [76]. The tablets with infill density of 20%, 35%, and 60% were successfully 3D printed and tested. As predicted, lower infill density resulted in faster dissolution. However, the micro-computed tomography imaging revealed that during the printing of the tablets with 20% infill, there was an issue with maintaining the internal structure geometry, which also manifested in higher deviations in the amount of dissolved itraconazole in the first 20 min of the dissolution test (Figure 10). The tablets with 60% infill were characterized by the slowest itraconazole release. This is directly connected with the difficulty of water penetration into the tablet due to the smaller pores and channels in the internal structure. Therefore, we chose 35% infill as the best formulation to evaluate the tablet shape, dose, and internal structure reproducibility in the 3D printing process. In all cases of 3D printed tablets, long-lasting supersaturation of the itraconazole was achieved. It is well-known that the persisting state of supersaturation may lead to bioavailability improvements, which is especially beneficial in terms of poorly soluble drugs such as itraconazole [77].

## 5. Conclusions

Our study has shown the detailed methodology for the development of immediate-release 3D printed tablets with liquid crystal-forming itraconazole. The development stage included both the optimization of the formulation composition and the correlation between the geometry of the printed object, namely the degree of infill, with shape reproducibility and drug dissolution.

The use of well-printable PVA polymer alongside the functionalized excipients, i.e., polyvinylpyrrolidone derivatives, during the hot-melt extrusion process covered not only the optimization of the mechanical properties of the filament and its printability but also the function of the polymer matrix in terms of intended drug release profiles. The results of the dissolution study and physicochemical analysis indicated that improved disintegration obtained due to the use of Kollidon^®^CL-M was more beneficial than the molecular rearrangement and liquid crystal phase transitions. The lower infill density favored faster dissolution of the drug from printed tablets.

Micro-computed tomography was utilized to confirm that the design of printed objects was properly reconstructed. The comprehensive analysis revealed that the infill density, which is often considered as a way to control or improve drug dissolution, should be utilized with a deep understanding of its effect on the 3D printed objects’ reproducibility. In the case of low infill densities, reproducibility issues, i.e., path disorder, increased layer dimension, and the path cohesion between cross-points, may occur. On the contrary, dense infill limits the surface area available for dissolution media and slows down the dissolution of the API.

In the case of the presented results, the most appropriate properties, i.e., good reproducibility during the object printing combined with superior drug dissolution, were achieved for the filament composed of 20% of itraconazole, 76% of PVA, and 4% of crospovidone acting as a disintegrant.

## Figures and Tables

**Table 1:** Composition of the filaments.

Formulation	Itraconazole	Poly(vinyl alcohol)	Copovidone	Crospovidone
PVA	20%	80%	-	-
PVA_K/VA		56%	24%	-
PVA_K/CL		76%	-	4%

**Table 2:** Hot-melt extruded filament characteristics.

Filament Composition	Diameter ± SD (mm)	Itraconazole Content ± SD (%)	Tensile Strength ± SD (MPa)	Young’s Modulus ± SD (MPa)
PVA	1.70 ± 0.02	19.67 ± 0.43	49.0 ± 10.3	2641.1 ± 144.4
PVA_K/CL	1.68 ± 0.07	19.60 ± 0.34	52.6 ± 19.8	2771.1 ± 347.2
PVA_K/VA	1.69 ± 0.05	19.20 ± 0.33	28.2 ± 7.1	2042.1 ± 256.3

**Table 3:** Comparison of values of T_g_, T_Sm-N_, and T_N-I_ of neat ITR and its mixtures with PVA, PVA_K/VA, and PVA_K/CL which were prepared in two forms: filament and 3DP tablet.

Sample	T_g_ (K)	T_Sm-N_ (K)	T_N-I_ (K)
Neat ITR	332	348	364
PVA filament	315	326	347
PVA 3DP tablet	306	330	344
PVA_K/CL filament	312	329	346
PVA_K/CL 3DP tablet	308	330	344
PVA_K/VA filament	317	328 (T_Sm-I_)	
PVA_K/VA 3DP tablet	315	330 (T_Sm-I_)	

**Table 4:** Parameters of 3D printed tablets.

Polymers	Infill (%)	Mass (mg)	Width (mm)	Length (mm)	Height (mm)	Number of Layers	Real Layer Height (mm)
PVA	35	252.82 ± 4.16	10.18 ± 0.03	20.15 ± 0.03	2.34 ± 0.03	16	0.142
PVA_K/VA	35	253.05 ± 3.67	10.08 ± 0.01	20.09 ± 0.01	2.89 ± 0.05	20	0.142
PVA_K/CL	35	250.12 ± 4.52	9.98 ± 0.02	19.85 ± 0.12	2.67 ± 0.03	17	0.154
PVA_K/CL	20	244.12 ± 5.77	9.96 ± 0.03	19.86 ± 0.09	3.65 ± 0.03	24	0.150
PVA_K/CL	60	239.73 ± 3.01	9.99 ± 0.05	20.05 ± 0.03	1.78 ± 0.02	11	0.158

**Table 5:** Comparison of µCT scan data of 3DP PVA_K/CL tablets with 20% (T_20), 35% (T_35), and 60% (T_60) infill ratio.

Description	Unit	T_20	T_35	T_60
Object volume	mm^3^	236	220	202
Percent object volume	%	33	41	60
Structure thickness	mm	0.25	0.20	0.19
Structure separation	mm	1.11	0.62	0.25
Volume of open pore space	mm^3^	485	312	134
Open porosity	%	67.2	58.5	39.9

**Table 6:** Comparison of µCT scan data of 3DP PVA_K/CL tablets with 35% of infill ratio.

Description	Unit	T_35_1	T_35_2	T_35_3
Object volume	mm^3^	220	224	213
Percent object volume	%	41	42	39
Structure thickness	mm	0.20	0.19	0.17
Structure separation	mm	0.62	0.62	0.62
Volume of open pore space	mm^3^	312	307	327
Open porosity	%	58.5	57.7	60.4

## Supplementary Materials

The following are available online at https://www.mdpi.com/1996-1944/13/21/4961/s1, Figure S1: 3D printed itraconazole-loaded tablet with 20% infill density; Figure S2: 3D printed itraconazole-loaded tablet with 35% infill density; Figure S3: 3D printed itraconazole-loaded tablet with 60% infill density.
