# Supplementary material for: Multivariate Design of 3D Printed Immediate-Release Tablets with Liquid Crystal-Forming Drug—Itraconazole
Source: Materials (Basel). 2020 Nov 4;13(21):4961. doi: 10.3390/ma13214961 (PMC7662355; doi:10.3390/ma13214961)
Supplement: Supplementary file 1 [file materials-13-04961-s001.pdf]

materials

MDPI

Supplementary Materials

Multivariate Design of 3D Printed Immediate-Release Tablets with Liquid Crystal-Forming Drug—Itraconazole

Witold Jamróz [1,*], Jolanta Pyteraf [1], Mateusz Kurek [1,*], Justyna Knapik-Kowalczuk [2,3], Joanna Szafraniec-Szczęsny [1], Karolina Jurkiewicz [2,3], Bartosz Leszczyński [4], Andrzej Wróbel [4], Marian Paluch [2,3] and Renata Jachowicz [1]

[1] Department of Pharmaceutical Technology and Biopharmaceutics, Jagiellonian University Medical College, Medyczna 9, 30-688 Krakow, Poland; jolanta.pyteraf@uj.edu.pl (J.P.); joanna.szafraniec@uj.edu.pl (J.S.-S.); renata.jachowicz@uj.edu.pl (R.J.)

[2] Division of Biophysics and Molecular Physics, Institute of Physics, University of Silesia, Uniwersytecka 4, 40-007 Katowice, Poland; justyna.knapik-kowalczuk@us.edu.pl (J.K.-K.), karolina.jurkiewicz@us.edu.pl (K.J.) marian.paluch@us.edu.pl (M.P.)

[3] Silesian Center for Education and Interdisciplinary Research, University of Silesia, 75 Pulku Piechoty 1a, 41-500 Chorzow, Poland

[4] Marian Smoluchowski Institute of Physics, Jagiellonian University, Łojasiewicza 11, 30-348 Krakow, Poland; bartosz.leszczynski@uj.edu.pl (B.L.), andrzej.wrobel@uj.edu.pl (A.W.)

* Correspondence: witold.jamroz@uj.edu.pl (W.J.), mateusz.kurek@uj.edu.pl (M.K.); Tel.: +48-12 62-05-600 (W.J. & M.K.)

Pictures of 3D printed itraconazole tablets with different infill density

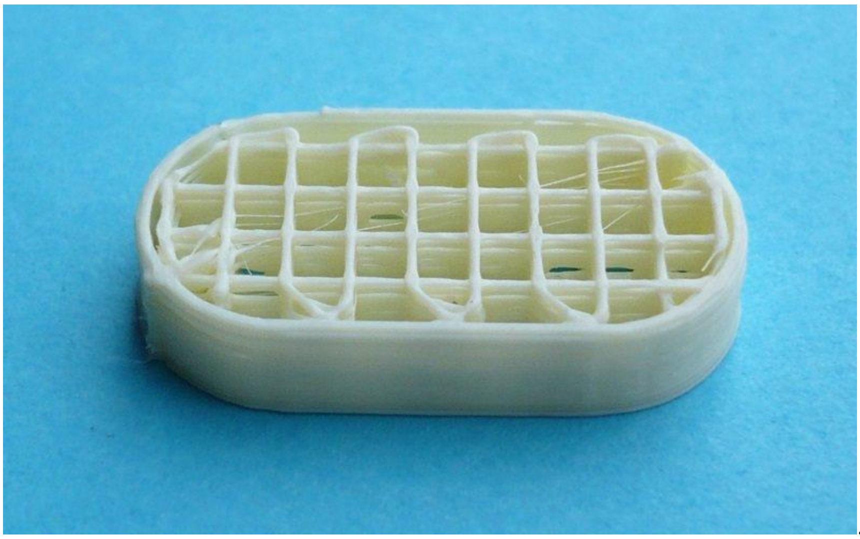

Figure S1. 3D printed itraconazole-loaded tablet with 20% infill density.

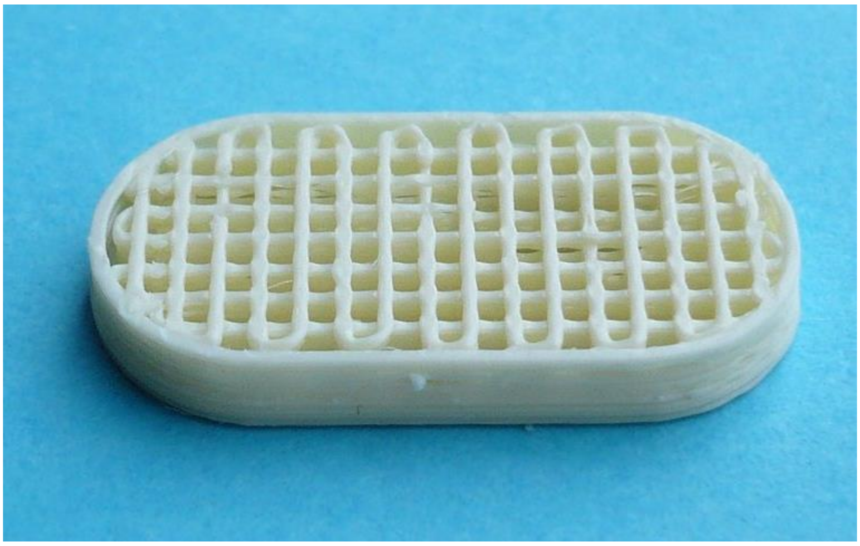

Figure S2. 3D printed itraconazole-loaded tablet with 35% infill density.

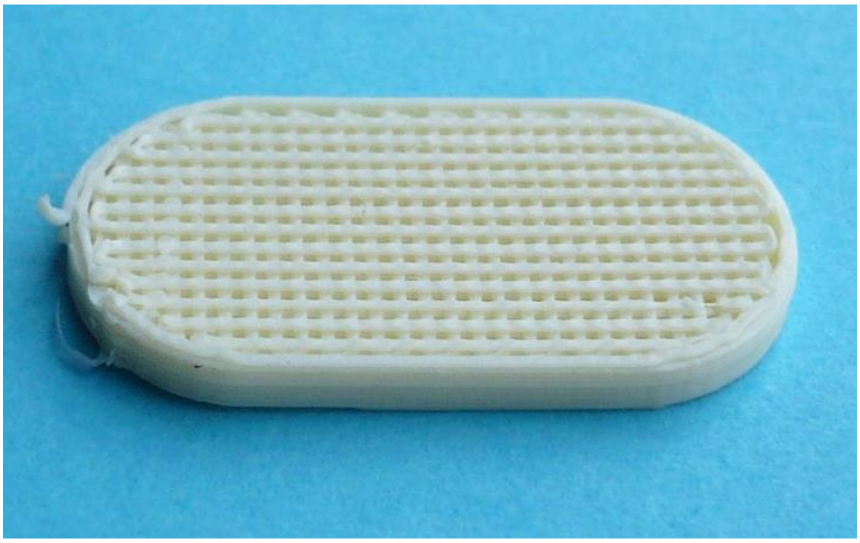

Figure S3. 3D printed itraconazole-loaded tablet with 60% infill density.

© 2020 by the authors. Submitted for possible open access publication under the terms and conditions of the Creative Commons Attribution (CC BY) license (http://creativecommons.org/licenses/by/4.0/).
